# Sex-stratified Correlates of Cyberbullying among Thai Adolescents: Insights from a School-based National Survey during COVID-19 Epidemic

**DOI:** 10.1007/s40653-025-00718-w

**Published:** 2025-06-04

**Authors:** Omid Dadras

**Affiliations:** Research Center for Child Psychiatry, University of Turku, Lemmkaisenkatu 3, 20520 Turku, Finland

**Keywords:** Cyberbullying, Adolescent health, Sex differences, Mental health, Risk behaviors

## Abstract

Cyberbullying is a significant concern among adolescents, impacting mental health and behavior. This study aimed to examine the sex-specific prevalence and correlates of cyberbullying victimization among Thai adolescents. Data from the 2021 Thailand Global School-based Student Health Survey, involving 5,657 students in grades 7–12, were analyzed. Cyberbullying victimization was assessed through self-reported experiences in the past 12 months. Twenty-three outcome variables across five domains (lifestyle, mental health, substance use, sexual behaviors, and violence) were examined. Logistic regression models, adjusted for age, assessed the associations between cyberbullying and these outcomes separately for males and females, accounting for the complex survey design and sampling weights. Approximately 17% of male and 13% of female students experienced cyberbullying in the past year. For males, cyberbullying was significantly associated with negative outcomes, including poor mental health (loneliness, insomnia due to anxiety, suicidal thoughts and attempts), increased substance use (cigarettes, alcohol, marijuana), and risky sexual behaviors (multiple sexual partners). Male victims were also more likely to engage in violent behaviors and experience physical attacks. For females, cyberbullying was linked to poor lifestyle choices (sedentary lifestyle, poor oral hygiene, inadequate fruit/vegetable intake), heightened mental health issues (loneliness, insomnia, suicidal thoughts and attempts), and substance use. Female victims were also at higher risk of experiencing violence and traditional bullying. This study documented high cyberbullying rates among Thai adolescents and calls for comprehensive mental‑health support, especially for males with elevated suicidal ideation; healthy‑lifestyle and social‑connectedness programs for females; and universal resilience and digital‑citizenship training for all youth.

## Introduction

Cyberbullying has emerged as a critical issue globally, affecting the mental health and well-being of adolescents (Zhu et al., 2021). it encompasses various forms of online harassment, including denigration, impersonation, exclusion, cyberstalking, and cyber threats (Kee et al., 2022). The emotional consequences of cyberbullying can be profound and far-reaching, with victims experiencing negative impacts even within their own homes due to the pervasive nature of online harassment (Anguyo et al., 2023). A recent systematic review of literature has suggested a strong association between cyberbullying victimization and poorer mental health outcomes in adolescents, such as increased risk of depression, anxiety, and suicidal ideation (Khalaf et al., 2023). Additionally, perpetrators of cyberbullying often experience high stress levels, poor school performance, and increased risks of depression and alcohol misuse (Gohal et al., 2023). While specific data on cyberbullying in Thailand is limited, global studies indicate that cyberbullying victimization and perpetration rates range from 14.6% to 52.2% among adolescents. The impact of cyberbullying on mental health may, however, differ between developed and developing economies, with factors such as access to technology, cultural norms, and support resources playing a role (Bansal et al., 2023). This wide range highlights the complexity of the issue and the need for a comprehensive understanding of the factors contributing to cyberbullying across different cultural and socioeconomic contexts.

In Asia, including countries like Thailand, the rapid adoption of smartphones and internet services among youth has paralleled the rise in cyberbullying incidents. Thailand provides a particularly instructive case study: as of January 2025, internet penetration in Thailand stood at 91.2 percent of its approximate 72 million population, and 68.3 percent of Thais were active social‑media users; levels comparable to peers such as Malaysia and Indonesia and other emerging economies in Latin America and Africa (DATAPORTAL, 2025). Smartphone ownership among Thai adolescents exceeds 90 percent, and nationwide digital schooling during COVID‑19 further intensified online exposure. The COVID‑19 pandemic prompted extended school closures and stay‑at‑home orders, resulting in a 25–35 percent increase in daily screen time among adolescents (Hedderson et al., 2023). Empirical studies report that cyberbullying incidents rose by approximately 20–30 percent during lockdowns, as isolated youth experienced and enacted more online aggression (António et al., 2024; Sorrentino et al., 2023). This digital integration has made adolescents particularly vulnerable to online harassment and abuse, leading to adverse psychological outcomes such as anxiety, depression, and, in severe cases, suicidal ideation (Sittichai, 2014). In Thailand, research on cyberbullying is still in its nascent stages, with limited studies exploring the prevalence and correlates of this behavior among adolescents. Available studies suggest that cyberbullying is a growing problem, with significant implications for the mental health and social well-being of Thai youth (Euajarusphan, 2021; Sittichai & Smith, 2020; Thumronglaohapun et al., 2022). A cross-sectional study conducted in 2020 across 14 schools and 4 universities in Thailand found that cyberbullying awareness and perception were high among both high school students and undergraduates. The study revealed that 29.6% of high school students and 39.6% of undergraduates had experienced cyberbullying victimization, primarily in the form of receiving mocking or rebuking messages (Thumronglaohapun et al., 2022). However, the majority of participants from the high schools were located in the northern and central regions of Thailand, which may limit the representativeness of the study results for the entire adolescent population in Thailand. Additionally, this study did not report on the correlates but rather assess the awareness and perception of cyberbullying. Other studies in Thailand mainly focused on university undergraduate students, typically representing youth older than 18 years old (Euajarusphan, 2021; Saengcharoensap & Rujiprak, 2021; Thongnopakun et al., 2023), and not representative at the national level (Chavanovanich et al., 2023; Euajarusphan, 2021; Sitthi et al., 2022; Sittichai & Smith, 2020). Besides, no previous study has comprehensively explored the impact of cyberbullying on healthy behavioral habits, risky sex behavior, violence, substance use, and food insecurity among Thai adolescents of the opposite sex. Moreover, the COVID-19 epidemic has further intensified this issue, as increased online activities during lockdowns and social distancing measures have heightened the exposure of adolescents to cyberbullying (Sorrentino et al., 2023; Thongnopakun et al., 2023). Despite the increasing trend, there is a notable research gap in understanding the sex-stratified prevalence and specific factors associated with cyberbullying in the Thai context during the pandemic.

Against this background, and building on evidence that boys and girls may experience and enact cyberbullying in distinct ways, with boys more often engaging in direct insults or threats and girls in relational behaviors such as exclusion and rumor‑spreading (Barlett & Coyne, 2014; Card et al., 2008; Duncan, 2006; Kowalski et al., 2014). This study aims to fill this gap by providing insights from a national school-based survey, examining the prevalence and correlates of cyberbullying among Thai adolescents with a focus on gender differences. Understanding these dynamics is crucial for developing targeted interventions and policies to mitigate the impact of cyberbullying and promote a safer online environment for all adolescents. We hypothesize that (1) adolescents who report being cyberbullied will show higher odds of adverse outcomes across multiple domains compared to those who are not cyberbullied, and (2) these associations will differ between male and female students, warranting gender-stratified analysis.

## Methods

### Data Source

We utilized data from the Thailand Global School-based Student Health Survey (GSHS) 2021, a comprehensive and nationally representative survey targeting students in grades 7–12, typically aged 13–17 years.

### Survey Design

This GSHS encompassed a wide range of health-related topics, including alcohol consumption, dietary habits, drug use, hygiene practices, mental health, physical activity, protective factors, sexual behaviors, tobacco use, and experiences of violence and unintentional injuries. Upon securing informed consent from participants, students were approached in their classroom settings. They were then provided with the questionnaire and asked to record their responses on a computer-scannable answer sheet. This method ensured efficient data collection and accuracy in capturing the students'health behaviors and experiences.

### Survey Sampling

The 2021 GSHS in Thailand utilized a two-stage cluster sampling method to ensure a thorough representation of students in grades 7–12 nationwide. In the first stage, schools were selected based on their enrollment size using probability proportional to size sampling, ensuring larger schools had a higher chance of selection. In the second stage, classes within these selected schools were randomly chosen, and all students in the selected classes were invited to participate. The survey achieved a remarkable school response rate of 92% and a student response rate of 90%, culminating in an overall response rate of 83%. In total, 5,657 students took part in the Thailand GSHS 2021 survey.

### Study Variables

*Independent variable (exposure):* Cyberbullying was defined by asking the question, “During the past 12 months, have you ever been cyberbullied (Count being bullied through texting, Instagram, Snapchat, Facebook, Twitter, TikTok, or other social media.)?” Responses were coded as 0 = no and 1 = yes.

*Dependent variables (outcomes):* Based on a comprehensive literature review and theoretical relevance, 23 outcome variables, potentially relevant to cyberbullying, were selected in five domains including lifestyle (five variables), mental health (four variables), current substance use (four variables), sex behaviors (three variables), violence (three variables), food insecurity (one variable), truancy (one variable), and social isolation (one variable). The details are presented in Table 1. In addition, participants self‑reported their sex with the question “What is your sex?” (coded 0 = male, 1 = female). We recognize that this item conflates biological sex and gender identity and does not capture transgender, non‑binary, or other diverse gender identities. Consequently, our analyses reflect comparisons between cisgender boys and girls only.

**Table 1:** Outcome variables definition, Thailand GSHS 2021

Domain	Variable	Question	Recode
Lifestyle	Sedentary lifestyle	During the past 7 days, how many days were you physically active for a total of at least 60 min per day?	1 = one or more days, 0 = zero days
	Poor oral hygiene	During the past 30 days, how many times per day did you usually clean or brush your teeth?	1 = less than one time a day, 0 = one or more times a day
	Inadequate fruit/vegetable	During the past 7 days, how many times did you eat vegetables, such as Chinese cabbage, water morning glory, Chinese kale, cucumber, and cabbage or how many times did you eat fruit, such as bananas, papaya, pineapple, guava, watermelon, mango, and oranges?	1 = less than one time a day, 0 = one or more times a day
	Drink soft drinks	During the past 7 days, how many times did you drink a can, bottle, or glass of a carbonated soft drink?	1 = at least one time, 0 = zero times
	Eat fast foods	During the past 7 days, how many days did you eat food from a fast food restaurant, such as a school canteen, hawker, or market?	1 = one or more days, 0 = zero days
Mental health	Often felt lonely	During the past 12 months, how often have you felt lonely?	1 = most of the time/often, 0 = never/rarely/sometimes
	Often insomnia due to anxiety	During the past 12 months, how often have you been so worried about something that you could not sleep at night?	1 = most of the time/often, 0 = never/rarely/sometimes
	Suicidal thoughts	During the past 12 months, did you ever seriously consider attempting suicide?	1 = yes, 0 = no
	Suicidal attempts	During the past 12 months, did you make a plan about how you would attempt suicide?	1 = yes, 0 = no
Current substance use	Cigarette	During the past 30 days, how many days did you smoke cigarettes?	1 = at least one day, 0 = zero days
	Other tobacco	During the past 30 days, how many days did you use any tobacco products other than cigarettes, such as roll-your-own tobacco or baraku?	1 = at least one day, 0 = zero days
	Alcohol	During the past 30 days, how many days did you have at least one drink containing alcohol?	1 = at least one day, 0 = zero days
	Marijuana	During the past 30 days, how many times have you used marijuana (also called weed)?	1 = one or more times, 0 = zero times
Sex behaviors	Ever had sex	Have you ever had sexual intercourse?	1 = yes, 0 = no
	had sex with ≥ 2 persons	During your life, with how many people have you had sexual intercourse?	1 = two or more, 0 = no sex or one person
	Not used condom in last sex*	The last time you had sexual intercourse, did you or your partner use a condom?	1 = yes, 0 = no
Violence	Physical attacked	During the past 12 months, how many times were you physically attacked?	1 = one or more times, 0 = zero times
	Physical fight	During the past 12 months, how many times were you in a physical fight?	1 = one or more times, 0 = zero times
	Seriously injured	During the past 12 months, how many times were you seriously injured?	1 = one or more times, 0 = zero times
	Traditional bullying	During the past 12 months, have you ever been bullied	1 = yes, 0 = no
Food insecurity	Often went hungry	During the past 30 days, how often did you go hungry because there was not enough food in your home?	1 = most of the time/often, 0 = never/rarely/sometimes
Truancy	Missed school without permission	During the past 30 days, on how many days did you miss classes or school without permission?	1 = at least one day, 0 = zero days
Social isolation	No close friend	How many close friends do you have?	1 = no close friend, 0 = at least one

### Statistical Analysis

Descriptive statistics were employed to describe the demographic characteristics of the participants. Bivariate chi-square test examined the differences in cyberbullying by sex; presented in Table 2 While age and gender both correlate with cyberbullying, we focus on gender-specific patterns due to well-documented differences in how boys and girls experience and enact online aggression. Age was controlled for rather than stratified because: 1) The GSHS sampling frame spans grades 7–12 but is designed to focus on early adolescence (ages 13–15). Within this relatively narrow developmental window, age‑related variation in cyberbullying prevalence is smaller and less consistent than gender differences (Biswas et al., 2022); 2) Stratifying by six grade‑levels would produce small cell sizes, particularly at the upper and lower ends, reducing power to detect meaningful associations; and 3) Interventions for cyberbullying are most often delivered by gender‑targeted curricula (e.g. relational‑aggression programs for girls, digital‑resilience training for boys). Controlling for age ensures we account for developmental effects without fragmenting our sample into impractically small age bands. Therefore, separate regression models, adjusted for age distribution, were conducted for male and female participants to assess the odds of selected outcome variables within each domain in relation to cyberbullying across opposite sexes. This method enabled the assessment of the sex-specific and independent impacts of cyberbullying on each selected outcome, irrespective of the possible confounding effects of age as documented in previous research (Barlett & Coyne, 2014). Consequently, it supports the development of gender-specific school-based interventions and preventive measures for students in Grades 7–12 and offers valuable insights for policy formulation (Kapitány-Fövény et al., 2022). The results were illustrated using forest plots, which displayed the odds ratios (OR) along with their respective 95% confidence intervals (95% CI). All analyses accounted for the effects of the multi-stage sampling design and sampling weights due to disproportionate sampling. The analysis was carried out using STATA 17 (Stata Corporation, College Station, Texas, USA), with a significance level set at 0.05.

**Table 2:** Descriptive characteristics and bivariate associations (cyberbullying × sex), Thailand GSHS 2021

	N (%)
Age group (years)	
< 14	1671 (22.9)
14–15	2182 (41.3)
≥ 16	1804 (35.8)
Sex	
Male	2504 (46.8)
Female	3135 (53.1)
Grade	
7 th	1743 (22.5)
8 th	905 (21.8)
9 th	1288 (21.1)
10 th	573 (12.3)
11 th	509 (11.4)
12 th	637 (10.8)
Total	5657 (100)
Cyberbullied *	
Male	392 (16.9)
Female	395 (13.3)

The difference between the opposite sex was significant at p= 0.017

## Results

### Sample Sociodemographic Characteristics

The Thailand GSHS 2021 survey included 5,657 students, with an age distribution of 22.9% under 14 years, 41.3% aged 14–15 years, and 35.8% aged 16 years or older. The gender distribution was 46.8% male and 53.1% female. Regarding educational grade, the participants were distributed as follows: 22.5% in 7 th grade, 21.8% in 8 th grade, 21.1% in 9 th grade, 12.3% in 10 th grade, 11.4% in 11 th grade, and 10.8% in 12 th grade. Approximately 17% of male and 13% of female students experienced cyberbullying in the past year.

### Correlates of Cyberbullying Among Male Adolescents

**Fig. 1:**
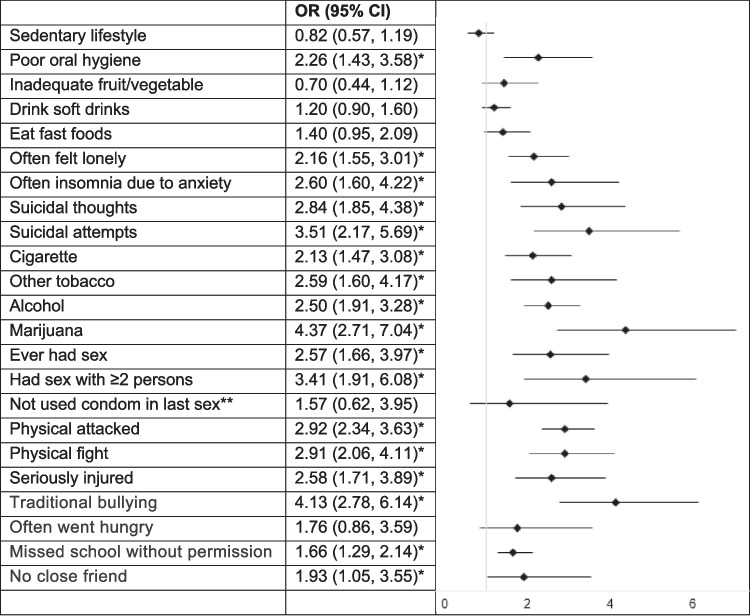
Odds of selected outcomes in male Thai students who experienced cyberbullying

The odds of selected outcomes for male Thai students who experienced cyberbullying are presented in Fig. 1. Cyberbullying was significantly associated with several adverse lifestyle, mental health, substance use, sexual behavior, violence, and social outcomes.

In terms of lifestyle, poor oral hygiene had increased odds (OR = 2.26, 95%CI: 1.43, 3.58), whereas other lifestyle factors like a sedentary lifestyle, inadequate fruit/vegetable intake, drinking soft drinks, and eating fast foods did not show significant associations. For mental health, cyberbullying was strongly linked to feelings of loneliness (OR = 2.16, 95%CI: 1.55, 3.01), insomnia due to anxiety (OR = 2.60, 95%CI: 1.60, 4.22), suicidal thoughts (OR = 2.84, 95%CI: 1.85, 4.38), and suicidal attempts (OR = 3.51, 95%CI: 2.17, 5.69). Substance use was significantly higher among cyberbullying victims, with increased odds for cigarette use (OR = 2.13, 95%CI: 1.47, 3.08), other tobacco products (OR = 2.59, 95%CI: 1.60, 4.17), alcohol consumption (OR = 2.50, 95%CI: 1.91, 3.28), and marijuana use (OR = 4.37, 95%CI: 2.71, 7.04). Sexual behaviors also showed significant associations, with higher odds of having ever had sex (OR = 2.57, 95%CI: 1.66, 3.97) and having had sex with two or more persons (OR = 3.41, 95%CI: 1.91, 6.08), although not using a condom during the last sexual encounter was not significant (OR = 1.57, 95%CI: 0.62, 3.95). Violence-related outcomes were notably higher, with increased odds of being physically attacked (OR = 2.92, 95%CI: 2.34, 3.63), engaging in physical fights (OR = 2.91, 95%CI: 2.06, 4.11), being seriously injured (OR = 2.58, 95%CI: 1.71, 3.89), and experiencing traditional bullying (OR = 4.13, 95%CI: 2.78, 6.14). Food insecurity was not significantly associated with cyberbullying (OR = 1.76, 95%CI: 0.86, 3.59), but truancy (OR = 1.66, 95%CI: 1.29, 2.14) and social isolation, such as having no close friend (OR = 1.93, 95%CI: 1.05, 3.55), were significantly higher among those who were cyberbullied.

### Correlates of Cyberbullying Among Female Adolescents

**Fig. 2:**
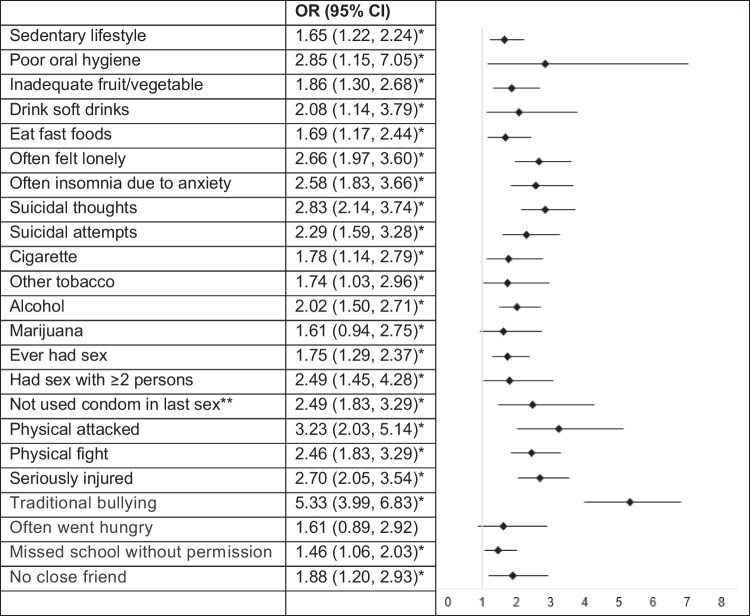
Odds of selected outcomes in female Thai students who experienced cyberbullying

The odds of selected outcomes for female Thai students who experienced cyberbullying are summarized in Fig. 2. Cyberbullying was significantly associated with several negative lifestyle, mental health, substance use, sexual behavior, violence, and social outcomes.

For lifestyle factors, female students who experienced cyberbullying had higher odds of leading a sedentary lifestyle (OR = 1.65, 95%CI: 1.22, 2.24), having poor oral hygiene (OR = 2.85, 95%CI: 1.15, 7.05), inadequate fruit/vegetable intake (OR = 1.86, 95%CI: 1.30, 2.68), drinking soft drinks (OR = 2.08, 95%CI: 1.14, 3.79), and eating fast foods (OR = 1.69, 95%CI: 1.17, 2.44). Mental health issues were notably higher among those who experienced cyberbullying, with increased odds of feeling lonely (OR = 2.66, 95%CI: 1.97, 3.60), having insomnia due to anxiety (OR = 2.58, 95%CI: 1.83, 3.66), experiencing suicidal thoughts (OR = 2.83, 95%CI: 2.14, 3.74), and making suicidal attempts (OR = 2.29, 95%CI: 1.59, 3.28). Substance use was also significantly associated with cyberbullying. The odds ratios for cigarette use (OR = 1.78, 95%CI: 1.14, 2.79), other tobacco products (OR = 1.74, 95%CI: 1.03, 2.96), alcohol consumption (OR = 2.02, 95%CI: 1.50, 2.71), and marijuana use (OR = 1.61, 95%CI: 0.94, 2.75) were all elevated among female cyberbullying victims. Regarding sexual behaviors, cyberbullying was associated with higher odds of having ever had sex (OR = 1.75, 95%CI: 1.29, 2.37), having sex with two or more persons (OR = 2.49, 95%CI: 1.45, 4.28), and not using a condom during the last sexual encounter (OR = 2.49, 95%CI: 1.83, 3.29). Violence-related outcomes were also higher among female students who experienced cyberbullying, including being physically attacked (OR = 3.23, 95%CI: 2.03, 5.14), engaging in physical fights (OR = 2.46, 95%CI: 1.83, 3.29), being seriously injured (OR = 2.70, 95%CI: 2.05, 3.54), and experiencing traditional bullying (OR = 5.33, 95%CI: 3.99, 6.83). Food insecurity did not show a significant association (OR = 1.61, 95%CI: 0.89, 2.92). However, truancy (OR = 1.46, 95%CI: 1.06, 2.03) and social isolation, such as having no close friend (OR = 1.88, 95%CI: 1.20, 2.93), were significantly higher among female students who experienced cyberbullying.

## Discussion

Cyberbullying among adolescents in Thailand has emerged as a significant concern, particularly after the COVID-19 pandemic (Thongnopakun et al., 2023). Recent studies indicate an alarming increase in the prevalence of cyberbullying among Thai youth (Chavanovanich et al., 2023; Kee et al., 2022; Saengcharoensap & Rujiprak, 2021). Our study found that approximately 17% of male and 13% of female students in Thailand experienced cyberbullying in the past year. These figures are much lower than those of recent studies in which cyberbullying ranged from 29% (Thumronglaohapun et al., 2022) to 45% (Cheyjunya, 2018) in high school students. This can be attributed to variations in the sampling methods, definitions, and measurements of cyberbullying, data collection methods, and timing of the survey across studies. However, our study used the data from Thailand GSHS 2021, a recent national school-based survey during the COVID-19 pandemic, with a standard and robust sampling methodology that ensures the representativeness of findings at the national level. Therefore, provides more precise estimates of the prevalence of cyberbullying at the national level following the pandemic, highlighting the pervasiveness of this issue among school-going adolescents in Thailand. Additionally, the observed gender differences in cyberbullying prevalence in our study can be attributed to cultural factors, particularly in Asian countries where males are more likely to be involved in cyberbullying perpetration (Sun et al., 2016). This is the first report reflecting upon these nuances among opposite in Thailand and can contribute to identifying at-risk populations and developing gender-specific interventions at Thai schools.

Our research also highlights distinct gender differences in the correlates of cyberbullying among Thai adolescents. For male students, cyberbullying was significantly associated with various negative outcomes, including poor mental health (feelings of loneliness, insomnia due to anxiety, suicidal thoughts and attempts), increased substance use (cigarettes, alcohol, marijuana), and risky sexual behaviors (having multiple sexual partners). Additionally, male victims of cyberbullying were more likely to engage in violent behaviors and experience physical attacks. Female students, on the other hand, exhibited different patterns. Cyberbullying among females was significantly linked to poor lifestyle choices (sedentary lifestyle, poor oral hygiene, inadequate fruit/vegetable intake), heightened mental health issues (feelings of loneliness, insomnia, suicidal thoughts and attempts), and substance use. Notably, female victims were also at higher risk of experiencing violence and traditional bullying.

While the association between cyberbullying and mental health issues (depression, anxiety, suicidal thoughts) is consistent across genders, aligning with broader research on cyberbullying effects (Alhajji et al., 2019a, 2019b; Kim et al., 2019; Levine et al., 2022), the observed differences in dietary and lifestyle choices and risk behaviors between genders may reflect societal expectations and peer influences unique to Thai culture (Cheung et al., 2020; Sitthi et al., 2022; Thumronglaohapun et al., 2022). Studies have shown that females may turn to unhealthy eating habits and be more prone to emotional eating or sedentary lifestyles as coping mechanisms for stress and anxiety caused by cyberbullying (Marco & Tormo-Irun, 2018). The association between cyberbullying and mental health issues, such as depression, anxiety, and suicidal thoughts, has been magnified during the COVID-19 pandemic due to heightened online presence and more exposure to cyberbullying (Kee et al., 2022; Marinoni et al., 2024). This contributed to lower self-esteem and increased feelings of loneliness, further deteriorating mental health (Alsawalqa, 2021). The social isolation and stress caused by the pandemic have made adolescents more vulnerable to the psychological impacts of cyberbullying (Sorrentino et al., 2023). The association between cyberbullying and mental health issues is further influenced by social media use and gender, with men experiencing greater cyberbullying victimization and perpetration when they have higher levels of social media use (Schodt et al., 2021). In addition, Male students'increased likelihood of engaging in violent behaviors and experiencing physical attacks could be related to societal norms that may encourage or tolerate aggression in males (Marcum et al., 2012). Nonetheless, the link between cyberbullying and poor mental health outcomes in both genders underscores the universal impact of online harassment on psychological well-being. Nonetheless, targeted strategies addressing gender differences in cyberbullying experiences and mental health outcomes are necessary.

In terms of violent experiences, the higher risk of experiencing traditional bullying, which may include physical violence, alongside cyberbullying suggests a potential overlap between online and offline victimization (Kowalski et al., 2012; Waasdorp & Bradshaw, 2015; Wang et al., 2019). This overlap suggests a potential link between online and offline victimization, highlighting the need for a comprehensive approach to intervention and prevention that addresses all forms of bullying (Cosma et al., 2019). Furthermore, the co-occurrence of traditional and cyberbullying is associated with a range of mental health problems, emphasizing the importance of addressing both forms of victimization in support programs (Waasdorp & Bradshaw, 2015). These programs should focus on developing social-emotional skills, promoting a positive school climate, and teaching digital citizenship (Cross et al., 2016). Additionally, the mental health issues associated with violent behaviors can contribute to fear-based absenteeism (Grinshteyn & Tony Yang, 2017). Unlike traditional bullying, cyberbullying can occur around the clock, potentially increasing the victim's desire to avoid school in fear of retaliation or further confrontation at school (Peebles, 2014). This may explain the higher rate of truancy among students who experienced cyberbullying in our study population and underscore the need for comprehensive anti-violence programs that address both cyberbullying and physical forms of aggression to effectively reduce truancy rates.

The observed association between cyberbullying and substance use in both genders among students in this study indicates a common coping mechanism, though the types of substances may differ (Alegría et al., 2018). The intentional and repeated nature of cyberbullying can lead to chronic emotional distress, potentially driving both genders to substance use as a coping strategy (Cénat et al., 2018; Gámez-Guadix et al., 2013; Livazović & Ham, 2019). Cyberbullying, especially when combined with traditional bullying, is also associated with increased binge drinking and marijuana use, with gender differences in these associations (Priesman et al., 2018; Williams et al., 2020) as documented in this study. Besides, males generally exhibit higher rates of cyberbullying perpetration, which may explain differences in the odds of substance use observed between genders (Sun et al., 2016) in our study, with male students having a higher likelihood. Cyberbullying can also lead to negative school experiences and lower academic performance, which may contribute to substance use as an escape mechanism (Schneider et al., 2012). In addition, the COVID-19 pandemic may have exacerbated the risk of substance use among young people, potentially amplifying the effects of cyberbullying on both genders (Layman et al., 2022).

The findings of this study underscore the need for gender-specific interventions and policies to address cyberbullying among adolescents in Thailand. Current evidence suggests that traditional anti-bullying programs may not adequately address the unique challenges posed by cyberbullying (Sittichai, 2014). We also acknowledge that our results derive from self‑reports, which can be influenced by gender norms around admitting distress or health behaviors. Consequently, intervention design should follow our observed associations rather than stereotypes: enhanced mental‑health screening and support must explicitly include male victims, given their higher reported odds of suicidal ideation and attempts (Mohammed Alhajji et al., 2019a, 2019b; Buelga Vásquez et al., 2022), while programs promoting healthy lifestyle habits and social connectedness may be especially beneficial for female victims (Buelga Vásquez et al., 2022). Both genders would benefit from universal components such as resilience training, bystander empowerment, and digital‑citizenship education that address the common correlates of cyberbullying (Kamaruddin et al., 2023). At the national level, policies should aim to create a safer online environment for adolescents. This includes implementing stricter regulations on social media platforms, promoting digital literacy, and ensuring that students have access to resources and support systems (Ang, 2015). Moreover, there should be an emphasis on training educators to recognize signs of cyberbullying and to provide gender‑inclusive support to all victims (Macaulay et al., 2018).

## Limitations

While this study provides important insights into the prevalence and correlates of cyberbullying among adolescents in Thailand, several limitations should be acknowledged. The cross-sectional design of the study limits the ability to draw causal inferences about the relationships between cyberbullying and various outcomes. Thus, while we report associations, such as between cyberbullying and poor oral hygiene, it is equally plausible that pre‑existing oral hygiene issues (which may affect peer perceptions and social standing) contribute to increased risk of being cyberbullied. This bidirectionality underscores that our findings reflect correlation, not causation. Longitudinal or experimental designs are needed to disentangle these pathways and to inform targeted intervention timing. The data were collected through self-reported measures, which are subject to biases such as social desirability and recall bias. Participants might underreport or overreport their experiences of cyberbullying and associated behaviors, leading to potential inaccuracies in the findings. Although the sample was nationally representative, the study relied on school-based surveys, which might exclude adolescents who are not attending school regularly or have dropped out. This could result in an underestimation of cyberbullying prevalence and its impacts, as these groups might have different experiences and vulnerabilities. In addition, the study focused on a specific period during the COVID-19 pandemic, which may have influenced the behaviors and experiences of adolescents in unique ways. The heightened online presence and stress during the pandemic could have exacerbated cyberbullying incidents and their impacts, and the findings might not fully generalize to non-pandemic times. Our primary exposure, cyberbullying victimization, was assessed via a single binary item (“During the past 12 months, have you ever been cyberbullied…?”). Although this aligns with standard GSHS methods, it may under‑capture the full spectrum of cyberbullying behaviors (denigration, impersonation, exclusion, stalking, threats) and does not differentiate by frequency or severity. Consequently, some adolescents who experienced less overt or less frequent forms of online harm may have answered “no,” leading to potential misclassification and underestimation of prevalence. Future studies should consider multi‑item instruments or behavioral checklists to capture nuanced dimensions of cyberbullying. Additionally, our survey’s binary sex item precluded identification of transgender or non‑binary adolescents, who prior research shows experience disproportionately high rates of cyberbullying (Holt et al., 2023). As a result, our sex‑stratified findings may not generalize to LGBTQ youth and could underestimate the overall burden of cyberbullying. Future studies should oversample gender‑diverse populations and include validated gender identity measures to better understand their unique vulnerabilities and needs. Lastly, the study did not explore all potential moderating factors, such as the role of family environment, peer support, school policies, or socioeconomic status, which could influence the relationship between cyberbullying and its outcomes. In particular, the GSHS lacks direct measures of household income or parental education, important determinants of both cyberbullying risk and adolescent well‑being (Tippett & Wolke, 2014), so our age‑ and sex‑adjusted models may suffer from residual confounding due to unmeasured SES disparities. Future research should consider these variables to provide a more comprehensive understanding of the factors that mitigate or exacerbate the effects of cyberbullying.

## Conclusion

This study highlights the significant impact of cyberbullying on the mental health and behavioral outcomes of Thai adolescents, revealing distinct gender differences in its correlates. The findings demonstrate that cyberbullying is associated with various negative outcomes, including mental health issues, substance use, and risky behaviors, with nuanced differences between male and female students. The COVID-19 pandemic has likely exacerbated these issues, increasing the vulnerability of adolescents to online harassment due to heightened internet use and social isolation. Acknowledging the limitations of this study, future research should adopt longitudinal designs, consider additional moderating factors, and explore the post-pandemic context to develop more targeted and effective interventions. Overall, the pervasive impact of cyberbullying underscores the urgent need for comprehensive strategies to address and mitigate its effects on adolescent well-being.

## Data Availability

The GSHS 2021 is a publicly available dataset and is available on the World Health Organization NCD Microdata Repository website at: https://extranet.who.int/ncdsmicrodata/index.php/catalog/
